# Bioenergetic modulators hamper cancer cell viability and enhance response to chemotherapy

**DOI:** 10.1111/jcmm.13642

**Published:** 2018-05-29

**Authors:** Diana Tavares‐Valente, Sara Granja, Fátima Baltazar, Odília Queirós

**Affiliations:** ^1^ Life and Health Sciences Research Institute (ICVS) School of Medicine University of Minho Campus de Gualtar 4710‐057 Braga Portugal; ^2^ Department of Sciences IINFACTS ‐ Institute of Research and Advanced Training in Health Sciences and Technologies CESPU, CRL University Institute of Health Sciences (IUCS) Gandra Portugal

**Keywords:** drug resistance, glioma, glycolytic inhibitors, tumour bioenergetic, warburg effect

## Abstract

Gliomas are characterized by a marked glycolytic metabolism with a consequent production of massive amounts of lactate, even in the presence of normal levels of oxygen, associated to increased invasion capacity and to higher resistance to conventional treatment. This work aimed to understand how the metabolic modulation can influence tumour aggressive features and its potential to be used as complementary therapy. We assessed the effect of bioenergetic modulators (BMs) targeting different metabolic pathways in glioma cell characteristics. The in vivo effect of BMs was evaluated using the chicken chorioallantoic membrane model. Additionally, the effect of pre‐treatment with BMs in the response to the antitumour drug temozolomide (TMZ) was analysed in vitro. Cell treatment with the BMs induced a decrease in cell viability and in migratory/invasion abilities, as well as modifications in metabolic parameters (glucose, lactate and ATP) and increased the cytotoxicity of the conventional drug TMZ. Furthermore, all BMs decreased the tumour growth and the number of blood vessels in an in vivo model. Our results demonstrate that metabolic modulation has the potential to be used as therapy to decrease the aggressiveness of the tumours or to be combined with conventional drugs used in glioma treatment.

## 1 BACKGROUND

During oncogenic transformation, tumour cells acquire metabolic features to sustain their proliferation and to create more robust subpopulations, adapted to the different microenvironmental conditions.^1^ The altered metabolism in cancer cells was first described in 1956, by Otto Warburg, who postulated that tumour cells rely mainly on glycolysis, instead of oxidative phosphorylation (OXPHOS).^2^ A reversion of the pH gradient across the cell membrane occurs with this event, being associated to some cancer hallmarks such as cell proliferation, invasion, metastasis and chemo‐ and radioresistance.^3^, ^4^ The high‐grade glioma subtype comprises anaplastic astrocytoma (World health organization (WHO) grade III) and glioblastoma multiform (WHO grade IV), being the last one the most aggressive, invasive and lethal subtype.^5^, ^6^ This type of tumour is characterized by a metabolic plasticity, with a higher dependence of glycolysis and consequent acidification of the tumour microenvironment by lactate/proton efflux.^7^, ^8^ The current available therapies present limited efficacy, leading to tumour relapse and poor patient survival rates.^5^ Temozolomide (TMZ) is a first‐line oral alkylating drug used in glioma treatment, being its cytotoxicity based on TMZ‐generated O6‐methylguanine‐DNA adducts. However, the DNA damage induced by TMZ can be repaired by the O6‐methylguanine‐DNA methyltransferase (MGMT) repair enzyme, which is associated with TMZ therapy resistance and treatment failure.^9^, ^10^ Therefore, it is important to develop more specific and effective therapies targeting glioma features, such as the reprogrammed metabolism.^11^ The glycolytic enzymes, specifically overexpressed in cancer cells, are one of the main targets in this field and several compounds targeting glycolysis are already in clinical trials.^12^ Dichloroacetate (DCA) is a pyruvate dehydrogenase kinase (PDK) inhibitor that redirects cell metabolism towards OXPHOS. PDK is a direct inhibitor of pyruvate dehydrogenase (PDH), a key enzyme that shifts the flux of pyruvate into mitochondria to promote OXPHOS. Many reports showed the promising effect of DCA in cancer therapy in in vitro and in vivo cancer models,^13^, ^14^, ^15^ although aspects such as its toxicity and dose limit effects are still unclear.^16^, ^17^ Other glycolytic inhibitor with potential anticancer activity is 2‐deoxy‐D‐glucose (2‐DG). 2‐DG is a glucose analogue that competes with glucose in the first step of glycolysis, being converted to deoxyglucose‐6‐phosphate, a molecule that cannot be further metabolized, inhibiting hexokinase 2 (HK2), thus blocking glycolysis and the pentose phosphate pathway.^18^ 2‐DG is described as being able to induce tumour cell death in different type of cancers.^18^, ^19^, ^20^, ^21^ Although the potential use of glycolytic inhibitors in cancer therapy, recent studies have demonstrated that in brain tumours, mitochondrial oxidation is also an important pathway in metabolism to support the rapid cell growth.^22^ Some studies have demonstrated that biguanides, used commonly in diabetes treatment and that act on OXPHOS, may also have antitumour action. Phenformin is an analogue of metformin that exhibits a larger antitumour activity in lung,^23^ breast 24 and colorectal cancers.^25^ Recently, it has been described that the compounds that target the mitochondria can also affect glycolysis and vice versa. For instance, metformin, which inhibits the complex I of the mitochondria respiratory chain, can also target HK2.^26^

Therefore, the aim of this study was to understand the importance of metabolic inhibition in glioma proliferation and aggressiveness, and how bioenergetic modulators (BMs), such as DCA, 2‐DG and phenformin, can be potentially used as antitumour drugs, namely as combined therapy. There are very few reports describing the metabolic behaviour of glioma cells under the conditions of this study, as well as the use of these metabolic modulators as coadjuvants of the standard treatment, TMZ.

## 2 MATERIALS AND METHODS

### 2.1 Cell lines and cell culture

U251 and SW1088, two high‐grade glioma cell lines, were obtained from American Type Culture Collection. The immortalized astrocyte cell line hTERT/E6/E7 HOXA9, previously retrovirally infected with MSCVneo vectors containing HOXA9 cDNA,^27^ was kindly provided by Professor Bruno Costa, ICVS, University of Minho. All cell lines were maintained in Dulbecco's Modified Eagle's Medium (DMEM) supplemented with 10% foetal bovine serum (FBS) and 1% penicillin‐streptomycin solution, at 37°C and 5% CO_2_.

### 2.2 Drugs

TMZ (Sigma‐Aldrich) was dissolved in dimethyl sulfoxide (DMSO) in a 100 mmol/L stock solution, from which the working solutions were prepared. The BMs 2‐DG, DCA and phenformin (Sigma‐Aldrich) were dissolved in PBS, to prepare stock solutions of 1000, 10 000 and 100 mmol/L, respectively, from which the working solutions were prepared.

### 2.3 Cell viability assay

Cells were plated into 96‐well plates, at a density of 3 × 10^3^ cells/well for all cell lines, for TMZ, 2‐DG and phenformin exposure, during 72 hours and DCA exposure, during 48 hours. After treatment, cell viability was determined by the sulforhodamine B (SRB) assay, as described previously.^28^ IC_50_ values were estimated from at least 3 independent experiments, each one in triplicate, using the GraphPad Software.

### 2.4 Colony‐forming assay

Five hundred cells were seeded in 6‐well plates and treated with the IC_50_ of the different BMs, during the respective incubation times. Untreated cells were used as control. After incubation, the medium containing the compounds was removed, cells were washed twice with PBS and then fresh medium was added. Cells were then allowed to grow for 10 days. The formed colonies were fixed for 5 minutes with 3.7% (w/v) paraformaldehyde in PBS and stained for 20 minutes with 0.05% (w/v) violet crystal in distilled water. The plating efficiency (PE) was calculated as the percentage of the number of grown colonies over the number of cells seeded in the control before BMs treatment. For each condition, the survival fraction was determined as the number of colonies over the number of cells seeded × 1/PE.

### 2.5 Metabolism assays (Extracellular glucose and lactate, and ATP content)

Cells were plated in 6‐well plates at a density of 3 × 10^5^ cells/well. Then, cells were treated with the respective BMs IC_50_, and the cell culture medium was collected after the respective incubation time, for glucose and lactate quantification. Glucose and lactate were quantified using commercial kits (SPINREACT), according to the manufacturer's protocols and normalized against total biomass. Untreated cells, incubated during the same period of time, were used as control. Results are expressed as total μg of metabolite/total biomass. Simultaneously, the cells were used for protein extraction and to quantify the intracellular ATP using a commercial kit (Molecular Probes), according to the manufacturer's instructions. The ATP content was normalized for the concentration of protein and also against the value obtained with untreated cells, incubated during the same period of time of the respective treatment, set as 1. The results presented correspond to the average of at least three independent experiments.

### 2.6 Wound‐healing assay

Cells were plated in 6‐well plates at a density of 1 × 10^6^ cells/well and the wound‐healing assay was performed. Cells were treated with different concentrations of BMs for 24 hours, and the wound areas were photographed at 0, 12 and 24 hours. The relative migration distances were analysed using Image J Software. The relative migration for glioma cells treated with the respective BM IC_50_ was compared with the control (untreated cells).

### 2.7 Invasion assay

Cell invasion in cancer cell lines was analysed using 24‐well BD Biocoat Matrigel Invasion Chambers, according to the manufacturer's instructions (354480, BD Biosciences). In brief, after matrigel invasion chamber rehydration with medium without FBS, cells were seeded and incubated with the IC_50_ of the different BMs for 24 hours. Then, the invading cells were fixed with methanol and stained with haematoxylin. Membranes were photographed in Olympus SZx16 stereomicroscope (16×), and invading cells were counted using the Image J software (version 1.41; National Institutes of Health). Invasion was calculated as percentage of cell invasion, normalized for the control condition.

### 2.8 Immunohistochemistry

Immunohistochemistry for Ki67 (AP10243CM, Gennova) was performed according to the avidin‐biotin‐peroxidase principle (R.T.U. Vectastin Elite ABC kit; Vector Laboratories), as previously described by our group.^6^ In brief, deparaffinized and rehydrated slides were submitted to heat‐induced antigen retrieval in the microwave for 15 minutes with 10 mmol/L citrate buffer (pH 6.0). After endogenous peroxidase inactivation, incubation with the primary antibody was performed for 2 hours at room temperature. The immune reaction was visualized with 3,3′‐Diamonobenzidine (DAB + Substrate System; Dako). All sections were counterstained with Gill‐2 haematoxylin. For negative controls, primary antibodies were replaced by a universal negative control antibody (N1699, Dako).

### 2.9 Chicken chorioallantoic membrane (CAM) assay

In brief, fertilized chicken eggs were incubated at 37°C. On day 3 of development, a window was made into the eggshell after puncturing the air chamber, and eggs were sealed with BTK tape and returned to the incubator. On day 9 of development, the U251 cell line suspension (2 × 10^6^ cells in 20 μL DMEM medium and matrigel (Corning ref. 354230)) was placed inside the eggs and then they were tapped and returned to the incubator. At day 13 of incubation, the control group received 40 μL of PBS and the treated group received 40 μL of 2x IC_50_ of each BMs. After 96 hours (day 17 of development), the chicken embryos were killed by placing them at −80°C for 10 minutes. CAMs with tumours were dissected, fixed in 4% paraformaldehyde at room temperature and included in paraffin for further analysis. Digital Images were taken on days 13 and 17 of development in a stereomicroscope (Olympus S2 × 16), using a digital camera (Olympus DP71). At the selected time‐points, the in ovo tumour perimeter was measured using the Cell B software (Olympus). Before paraffin inclusion, tumours were photographed ex ovo for blood vessel counting. The number of blood vessels was counted using the Image J software. An additional group of eggs (n = 20) was used to evaluated the direct effect of BMs on the CAM and chicken embryo.

### 2.10 Effect of the bioenergetic modulators on TMZ cytotoxicity

1.5 × 10^3^ cells/well were seeded into 96‐well plates and pre‐treated with a fixed concentration of BMs (a concentration previously determined that increase cytotoxicity of TMZ but do not induce cell death per se), during the respective incubation times. In untreated cells, the medium was replaced at this time‐point. After the period of incubation, the medium containing the compounds was removed, cells were washed twice with PBS and the cells pre‐treated and not pre‐treated with BMs were exposed to TMZ at the same range of concentrations previously used for 72 hours. The effect of TMZ alone and BM + TMZ on cell viability was evaluated using the SRB assay. Additionally, the action of BMs on TMZ effect on cell migration and colony formation ability was evaluated through wound‐healing and colony formation assays, respectively. In both assays, cells were pre‐treated or not with a fixed concentration of BMs (2‐DG (5 mmol/L), DCA (20 mmol/L) and phenformin (0.01 mmol/L)—concentrations previously determined that increased cytotoxicity of TMZ but did not induce cell death per se) for the respective time of incubation. After this period of incubation, cells were treated with 100 μmol/L of TMZ for 72 hours in the colony formation assay and until 48 hours in the wound‐healing assay. The assays continued as described previously in this materials and methods section.

### 2.11 Statistical analysis

The GraphPad prism 5 software was used, with the Student's *t*‐test, considering significant values to be *P *≤* *.05.

## 3 RESULTS

### 3.1 Treatment with bioenergetic modulators affects cell survival and changes the metabolic profile of glioma cells

**Figure 1:**
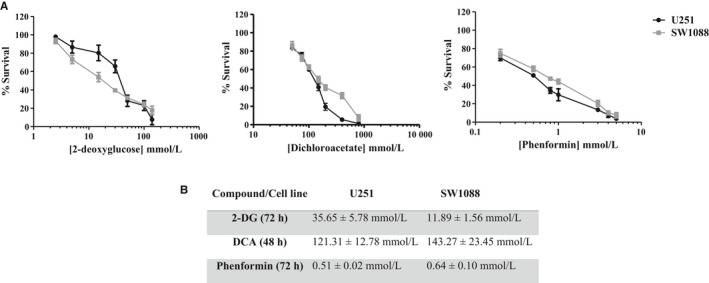
Effect of bioenergetic inhibitors (BMs), 2‐DG, DCA and phenformin on total biomass of glioma cells, U251 and SW1088. A, Cell survival was assessed by the sulforhodamine B assay. B, The IC_50_ values determined after the respective incubation time. Results represent the mean ± SEM of triplicates from at least three independent experiments

**Figure 2:**
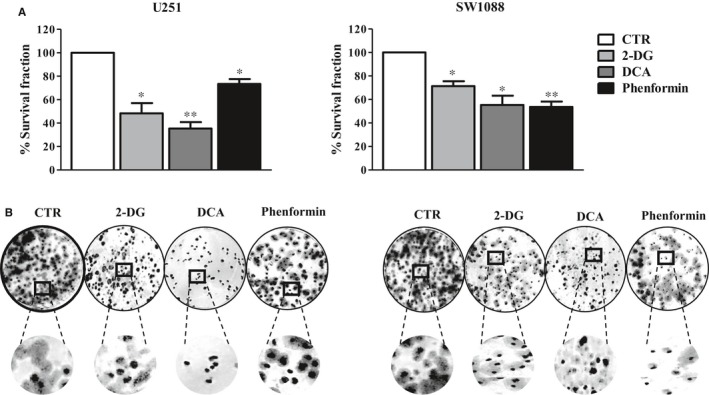
Effect of DCA, 2‐DG and phenformin on cell colony formation. A, Glioma cells were incubated with different compounds, and after a period of recovery for 10 days without compounds, the survival fraction was calculated. B, Representative pictures of colony formation in both cell lines. Pictures were taken at 200× magnification in a Nikon eclipse TE 2000‐U microscope. Results represent the mean ± SEM of duplicates from three independent experiments. *P <* *.05; **P <* *.01; compared to untreated cells

**Figure 3:**
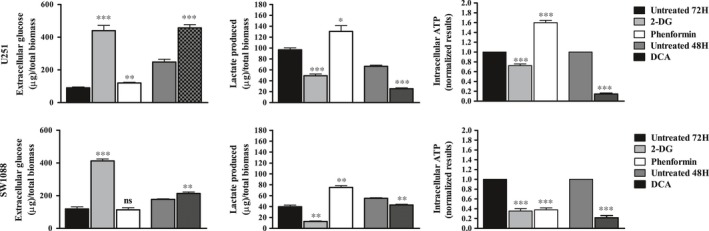
Metabolic profile of glioma cells estimated by extracellular glucose (A) and lactate (B) and ATP production (C), after treatment with 2‐DG, DCA and phenformin. Cells were incubated in the presence of the IC_50_ of the compounds, at the respective time of incubation. After this time, the metabolic parameters were quantified. Glucose and lactate levels were normalized against the biomass content. Untreated cells were used as control. ATP levels were normalized against the protein content of the extract, and against the value obtained with untreated cells, set as 1. Results are presented as mean ± SEM in triplicate of at least three independent experiments. *P <* *.05; **P <* *.01; ***P <* *.001 compared to untreated cells. Ns, no significant

Cell behaviour of two glioma cell lines (U251 and SW1088) was evaluated on metabolic remodelling using different metabolic modulators, 2‐DG, DCA and phenformin. A decrease in cell viability was triggered in both cell lines by the three compounds, in a dose‐ and time‐dependent manner (Figure 1A). The lower IC_50_ values were found for phenformin (<1 mmol/L for both cell lines), whereas 2‐DG IC_50_ values were in the range of 10‐35 mmol/L and DCA IC_50_ values were higher than 100 mmol/L (Figure 1B). Accordingly, all the compounds induced a decrease in the ability of both cell lines to form colonies, after a recovery period in the absence of compounds (Figure 2A). In U251 cells treated with 2‐DG and DCA, not only the colony number was lower but also the colony size. In contrast, the phenformin effect in the decreased ability to form colonies was more noticeable in SW1088, although the effect in the size was not so evident (Figure 2A,B). As referred previously, tumour cells present a metabolic reprogramming, being most of cellular ATP generated from glucose via aerobic glycolysis (“Warburg effect”) rather than by OXPHOS, what leads to a high rates of lactic acid production. However, most tumours do not depend completely of glycolysis for ATP supply, as mitochondrial metabolism is not decreased in all cancer cells.^29^ To analyse the metabolic profile in glioma cells, intracellular ATP content and extracellular lactate and glucose levels were measured for both cell lines treated with the respective BM IC_50_ values. Untreated cells, incubated for the same period of time, were used as control. It was observed that the glycolytic inhibitors, 2‐DG and DCA were able to reduce the consumption of glucose, as well as the production of lactate and ATP content, in both cell lines, although more evident for U251 cells, especially for DCA (Figure 3). Different results were obtained when the glioma cells were treated with phenformin. In U251 cell line, a significant increase in glucose and lactate extracellular levels was observed, as well as ATP production, in opposition to what happened with the other BMs. This effect can be due to an activation of energy production through glycolytic pathway when OXPHOS was inhibited. In SW1088 cells, glucose consumption was not altered and lactate production was higher compared to the control. Concerning ATP content, the blockage of this pathway impaired the energy production by this cell line. This indicates that SW1088 present a markedly oxidative phenotype, as inhibition by phenformin induced the most relevant alterations in the metabolic profile of this cell line. In contrast, U251 cells present a more glycolytic phenotype, as the glycolytic inhibitors induced a higher decrease in glucose consumption, and lactate/ATP production.

### 3.2 Metabolic modulation reduces the migration and invasion capacity of glioma cells

**Figure 4:**
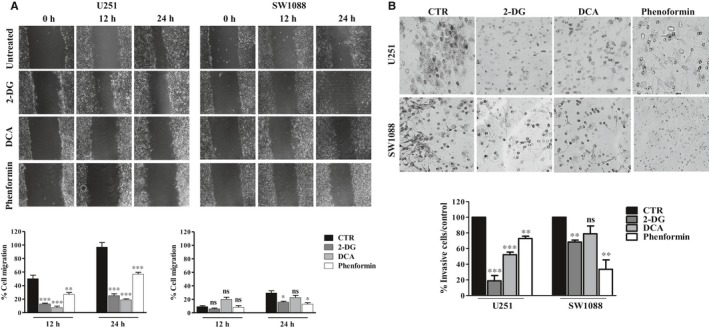
Cell migration (A) and cell invasion (B) of glioma cell lines after 2‐DG, DCA and phenformin treatment. Cell migration and invasion were evaluated by the wound‐healing and matrigel assays, respectively, after treatment with the compound IC_50_ values. Pictures were taken at 40× magnification (migration) and 200× magnification (invasion) in a Nikon eclipse TE 2000‐U microscope. Results represent the mean + SEM of at three independent experiments. *P <* *.05; **P < .01 compared to untreated cells (control). Ns, no significant

To understand the influence of metabolic modulation on the motility of glioma cell lines, we assessed cell migration by the wound‐healing assay, 12 and 24 hours after the treatment with BMs. Concerning U251 cells, all metabolic inhibitors induced a decrease in the motility, compared to untreated cells (Figure 4A). Nevertheless, phenformin was the less effective in this reduction. In SW1088 cells, metabolic modulation was not able to reduce the migratory ability in a significant way, as it presented already a low ability to close of wound, after 12 hours. However, after 24 hours, 2‐DG and phenformin induced a reduction in cell migration capacity. It was also observed that the invasion ability was affected by metabolic modulation (Figure 4B). This inhibition was more evident in U251 cells, with the glycolytic modulators, as they present a higher basal invasion capacity, compared to SW1088 cells. Concerning phenformin, it was not able to induce a significant decrease in invasion. Concerning SW1088 cells, phenformin induced a higher reduction in the invasive capacity, compared with glycolytic inhibitors, namely DCA.

### 3.3 Treatment of cancer cells with the metabolic modulators decreases glioblastoma proliferation in vivo

**Figure 5:**
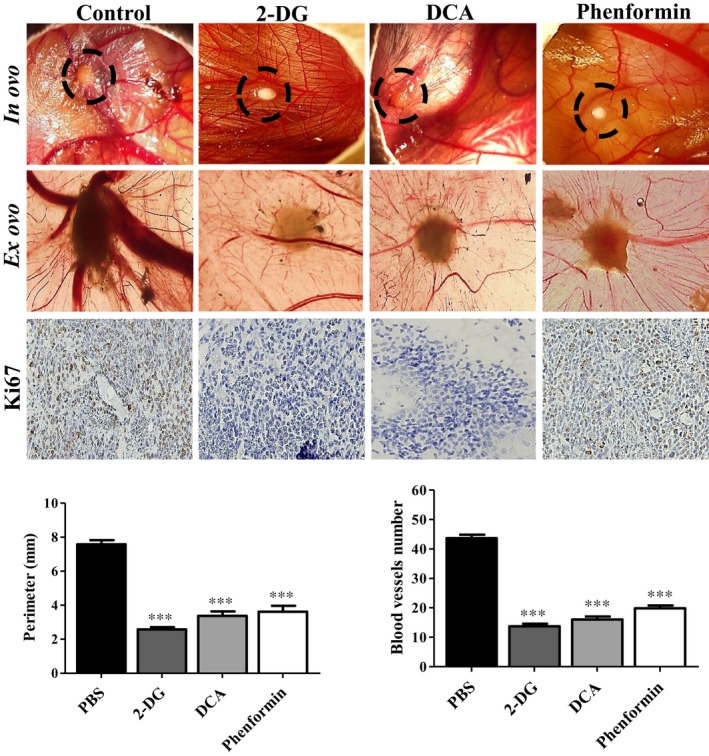
In vivo effect of BMs in U251 glioma microtumour growth. Representative pictures (16× [in ovo] and 12.5× [ex ovo] magnifications) of BMs effect on the perimeter and in vascularization of tumours, after 4 days of treatment in and ex ovo. Tumour growth was measured in ovo, and blood vessels around the tumours were counted ex ovo. Immunohistochemical analysis of Ki67 expression in treated tumours was compared with treated group (200× magnification). Pictures are representative of n = 20 eggs. ***P <* *.001 compared to untreated cells

According to our in vitro results, U251 cells exhibit higher glycolytic rates, compared with SW1088 cells. However, all the compounds including phenformin were able to decrease cell migration/invasion and cell proliferation of these cells. Therefore, we aimed to evaluate the efficacy of these compounds in an in vivo chicken chorioallantoic membrane (CAM) model. U251 cells were grown in the CAM of chicken embryos for 4 days, and treatment with 2X IC_50_ values of the three BMs was performed during 4 days. As demonstrated in Figure 5, all the BMs induced a decrease in tumour size, compared to the untreated group. The tumour perimeter of treated microtumours was around 3 or 4 mm, whereas one of the controls (untreated) was about 7 mm. Furthermore, BM treatment also reduced the number of blood vessels formed around the tumour. The number of tumour vessels in treated groups was about 10 or 15 vessels and in the control group was about 45 vessels (Figure 5). Additionally, tumour cell proliferation was reduced, compared to control, shown through Ki67 expression (Figure 5), being the glycolytic inhibitors the compounds that induced a higher decrease in tumour proliferation. These results demonstrated BMs ability to reduce the tumour cell population and the vascular support. It is important to notice that none of the metabolic inhibitors induced a decrease in blood vessels and chicken embryo viability when we tested in the CAM without tumour, under the same conditions (Figure S1A,B).

### 3.4 Metabolic inhibition potentiates temozolomide cytotoxicity

**Figure 6:**
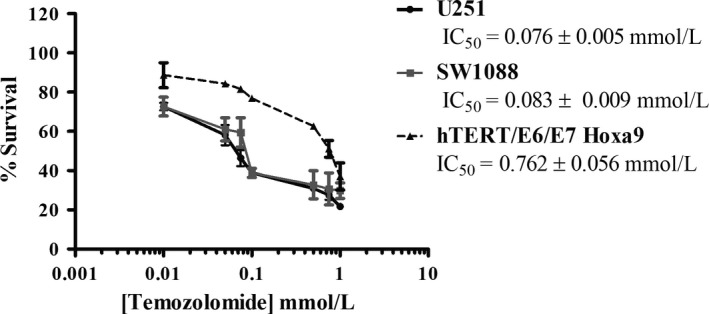
Effect of TMZ on total biomass of three cell lines, after 72 h of exposure assessed by the sulforhodamine B assay. Results represent the mean ± SEM of triplicates from at least three independent experiments

**Figure 7:**
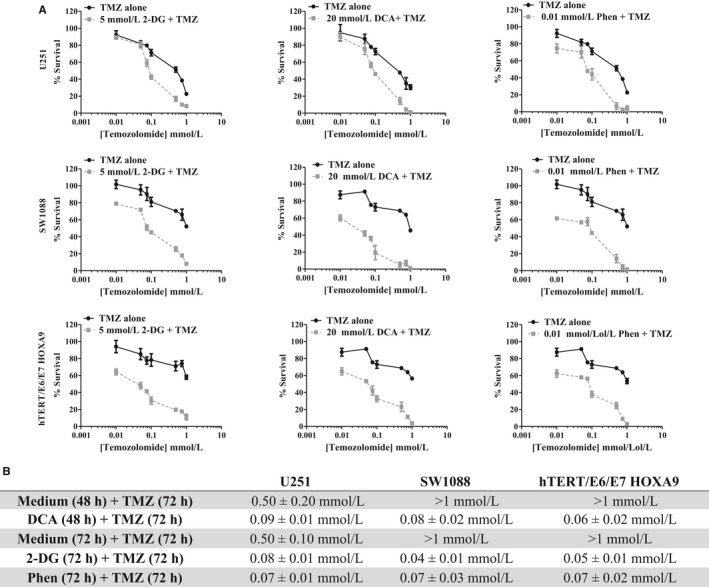
Effect of BMs, 2‐DG (5 mmol/L), DCA (20 mmol/L) and phenformin (Phen. 0.01 mmol/L), pre‐treatment on TMZ cytotoxicity, in U251, SW1088 and hTERT/E6/E7 HOXA9 cells. Cells were pre‐treated or not with fixed concentration of BMs during the respective incubation time, followed by the incubation with increasing concentrations of TMZ (0.01‐1 mmol/L). A, Cell survival was assessed by the sulforhodamine B assay. B, IC_50_ values, determined after the respective incubation time. Results represent the mean ± SEM of triplicates from at least three independent experiments

**Figure 8:**
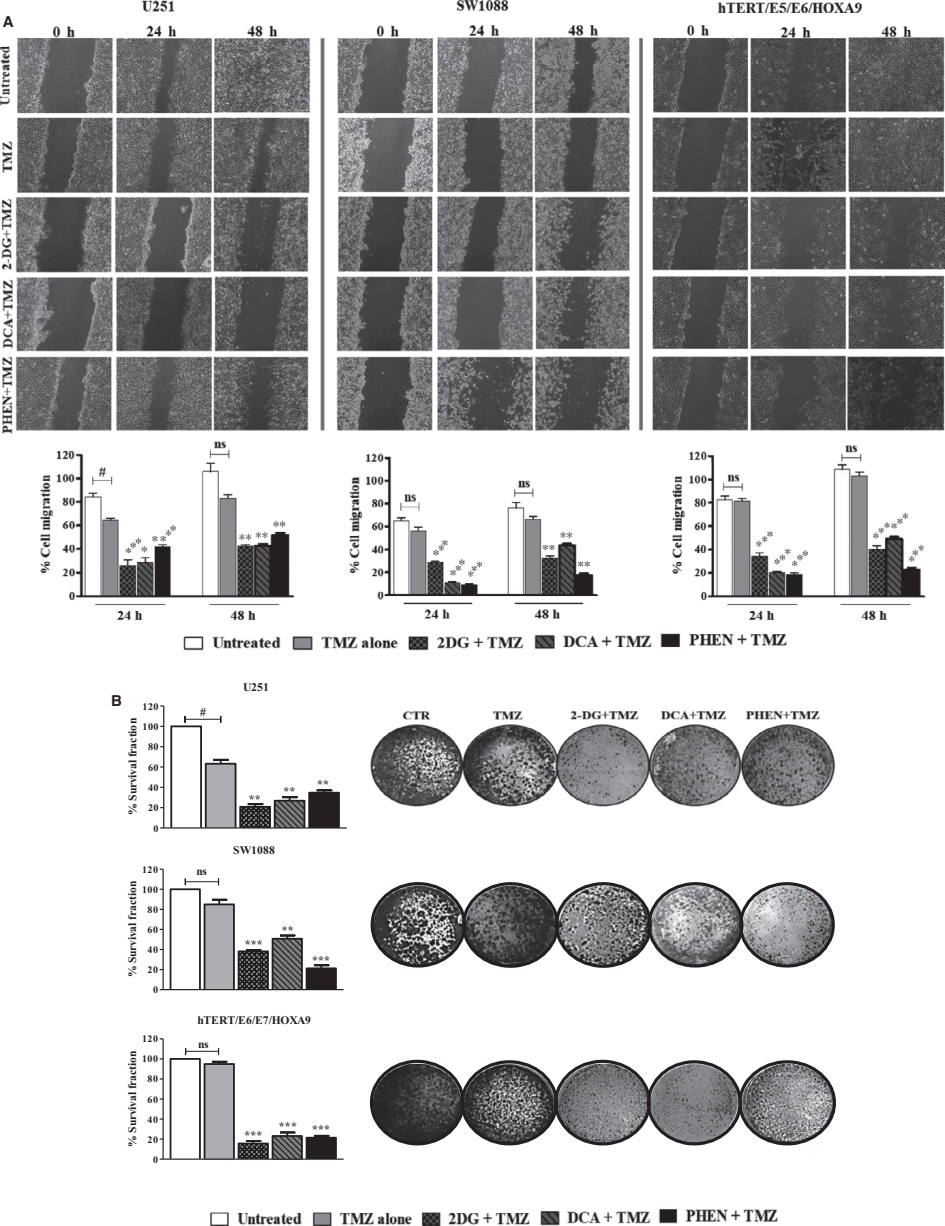
Effect of 2‐DG (5 mmol/L), DCA (20 mmol/L) and phenformin (Phen. 0.01 mmol/L), pre‐treatment on TMZ effect on cell migration and cell colony formation. All the cell lines, U251, SW1088 and hTERT/E6/E7 HOXA9 cells were pre‐treated or not with fixed concentrations of BMs during the respective incubation time, followed by the incubation with 100 μmol/L of TMZ. A, Cell migration was quantified by the wound‐healing assay, after pre‐treatment with the BMs and during incubation with TMZ. Pictures were taken at 40× magnification (migration) and 200× magnification (invasion) in a Nikon eclipse TE 2000‐U microscope. B, Representative pictures of colony formation in both cell lines. Cells were incubated with different compounds, and after a period of recovery for 10 days without compounds, the survival fraction was calculated. Pictures were taken at 200× magnification in a Nikon eclipse TE 2000‐U microscope. Results represent the mean + SEM of at three independent experiments. *P <* *.05; **P <* *.01; ***P <* *.001 compared to cells treated with TMZ alone. ^#^ *P *<* *.05; compared to untreated cells (control) Ns, no significant

This study also aimed to investigate the influence of BMs, acting at different metabolic targets, on the efficacy of the conventional antitumour drug TMZ, with the objective to overcome the treatment resistance commonly developed during therapeutic regimens. As described previously, the main mechanism of resistance in glioma is increased MGMT activity. Both glioma cell lines used did not express this enzyme, confirmed by quantitative PCR (Figure S2) and also reported in the literature.^30^, ^31^ For that reason, we decided to include another cell line from human astrocytes: the hTERT/E6/E7 HOXA9 cell line, transfected with the HOXA9 gene, described as presenting an aggressive behaviour and treatment resistance.^27^ This cell line showed a higher expression of MGMT gene compared to glioma cell lines (U251 and SW1088). The HOX genes are expressed in many human cancers, being responsible for different oncogenic features, including cell proliferation, migration and metastization and resistance to treatment, namely in gliomas.^32^, ^33^, ^34^ Firstly, we evaluated the cytotoxic effect of TMZ alone in different cell lines. Accordingly, and after 72 hours of treatment, it was observed that hTERT/E6/E7 HOXA9 cell line presented the highest TMZ IC_50_ (762.08 μmol/L) value (Figure 6), compared also to the parental cell line of astrocytes non‐transfected with HOXA9.^27^ The other two cell lines, U251 (IC_50_ = 75 μmol/L) and SW1088 (IC_50_ = 85 μmol/L), presented lower IC_50_ values. Trying to correlate the bioenergetic status of the cells with chemoresistance in cancer treatment, we evaluated the effect of cell metabolism inhibition on TMZ cytotoxicity. To evaluate the combined effect of BMs plus TMZ, all the three cell lines were pre‐treated with each BM and then exposed to different concentrations of TMZ, during 72 hours. As Figure 7 demonstrates, all the BMs enhanced TMZ cytotoxicity. 2‐DG, DCA and phenformin were able to potentiate the drug action, even when combined with lower concentrations of TMZ (between 0.01 and 0.1 mmol/L). According to these results, pre‐treatment with BMs, before incubation with TMZ, decreased also the cell migration capacity and the ability to form colonies, comparing to cells treated only with TMZ (Figure 8A,B, respectively). Overall, our results showed that all the compounds increase the cytotoxicity of TMZ as well as its effect on inhibiting cell migration, namely for the glycolytic inhibitors in U251 cells and phenformin in SW1088 and hTERT/E6/E7 HOXA9 cells. This increase in TMZ toxicity is more evident in hTERT/E6/E7 HOXA9 cells, which was characterized as the less sensitive cell line to this drug. Furthermore, we showed that all BMs, induced per se a decrease in cell survival and alterations in the cell metabolic profile, decreasing glucose consumption as well as lactate and ATP production, also in hTERT/E6/E7 HOXA9 cells, similarly to what happened with U251 and SW1088 cell lines, being this effect more noticeable for the glycolytic modulators (Figure S3). Additionally, all the compounds decrease the migration and colony formation ability in this cell line, approximately in the same extent to what happened with the other cell lines, showing that hTERT/E6/E7 HOXA9 is more resistant to TMZ than U251 and SW1088 glioma cell lines, but the effect of the BMs was similar in all of them (Figure S4).

## 4 DISCUSSION

Although the knowledge of the influence of the bioenergetic status on tumour characteristics increased greatly in the last years, there is a relatively modest knowledge on the efficacy of metabolic inhibitor compounds, namely in the clinical setting. One of the most aggressive and lethal types of brain human cancer is glioblastoma. However, the efficacy of the current therapies is very modest due to the development of multidrug resistance (MDR) phenotype, together with the disease recurrence.^30^ The switch of metabolism present in gliomas, with an increase in glycolysis as main energy source, is correlated with a worse prognostic and failure of antitumour therapies.^7^

In this study, we intend to understand the role of the reprogrammed metabolism and how its modulation can improve the conventional existent therapies. Many reports showed that the use of metabolic inhibitors potentiate the antitumour therapy and reduce tumour aggressiveness 35, 36, 37 Despite the existence of many compounds that target the altered metabolism, we decided to use different compounds, 2‐DG, DCA and phenformin, which target different steps of the metabolic network on tumour cells. 2‐DG inhibits glycolysis, targeting HK and also competes with the entrance of extracellular glucose into the cells by GLUTs.^38^ Other reports showed the effect on tumour cells induced by this compound alone or in combination with other drugs.^18^, ^19^, ^20^, ^21^ However, few reports showed the effectiveness of 2‐DG in glioma therapy. The pyruvate mimetic, DCA, redirects the tumour metabolism from glycolytic pathway to OXPHOS, inducing a decreasing the mitochondrial membrane potential and activating the K^+^ channel Kv1.5.^11^, ^39^ DCA is normally used to treat human hereditary mitochondrial metabolic diseases and lactic acidosis, but it has been recently evaluated in several pre‐clinical cancer therapies including prostate,^40^ colon 41 and breast cancer.^42^ DCA safety and efficacy has been studied in glioblastoma patients in a clinical trial (NCT01111097), but no results are published until now. Although most of the new metabolic antitumour agents target the glycolytic metabolism, we also assayed phenformin, a biguanide that showed promising results in the fight against cancer.^43^, ^44^ The main mechanism to explain the biguanide anticancer effect is the inhibition of the mitochondrial complex I, with a subsequent overproduction of reactive oxygen species (ROS).^45^ Additionally, these drugs activate 5′‐AMP‐activated protein kinase (AMPK) that inhibits mammalian target of rapamycin complex 1 (mTORC1) leading to reduced cell proliferation. Recently, some studies have demonstrated that biguanides may also have antitumour action and enhance the efficacy of TMZ treatment in glioma cells and glioma stem cells.^46^, ^47^ Nevertheless, most of these studies used metformin, which less potent than phenformin.^37^ For that reason, more studies on its effect on glioma therapy are needed.

As anticipated, in the present study, cell treatment with the energetic modulators changed the metabolic parameters, in most of the conditions used. Based on our findings, we hypothesized that the glycolytic pathway seems to be the main source of energy in U251 cells, as treatment with phenformin did not induce a depletion of cellular ATP. It is important to notice that the biguanide compounds, such as phenformin, require a functional OXPHOS to inhibit the complex I and consequently decrease ATP levels in the cell.^48^ In contrast, treatment with the antiglycolytic agents reduced significantly the cell ATP content and inhibited, in a greater extent, lactate production and glucose consumption. This can be explained by a mitochondrial dysfunction in this cell line, suggesting that forced utilization of defective OXPHOS could be toxic to the cells. Regarding SW1088 cells, our results indicate that both glycolysis and OXPHOS contribute to energy production. Indeed, all the agents led to a decrease in the ATP content in a similar way. About the glycolytic inhibitors, the effect was greater when 2‐DG was used, what is particularly evident in the glucose levels in the culture medium. Concerning phenformin, SW1088 cells were able to rely on glycolysis when the OXPHOS is inhibited, as glucose consumption was maintained in levels similar to the control and lactate production was higher. However, ATP production was lower, revealing that glycolysis is less effective as energy source. Moreover, we found that metabolic inhibition can also decrease the migration/invasion abilities of tumour cells, important hallmarks in the first steps of the metastatic process. We can hypothesize that the metabolic inhibition reduced lactate production and consequently decreased lactate and proton efflux, increasing extracellular pH (pHe), and motility is compromised. Our research group showed that blockage of lactate efflux, through MCT inhibition decreased the migration and invasion abilities in different cancer models.^6^, ^49^ Additionally, we observed that metabolic inhibition, by the use of glycolytic inhibitors, induced an increase in pHe compared to untreated cells (Table S1). Nevertheless, our results also demonstrated that when the cells were exposed to phenformin, the production of lactate was high and the pHe decreased. However, these in vitro assays do not mimic all the in vivo conditions for the migratory and invasive characteristics, and other processes not evaluated in this work, can be involved in the decrease in migratory and invasive capacity of cells. To support the previous results, we used an in vivo model, the CAM assay, where we observed a decrease in tumour proliferation and the number of surrounded vessels when treated with BMs. CAMs without tumours were exposed to the same concentrations of BMs and no toxic alterations were observed. In fact, few reports demonstrated toxic effects in normal cell lines or tissues induced by these metabolic inhibitors. Cheng et al^20^ detected some brain toxicity using 2‐DG; however, to the best of our knowledge, no others reports referred adverse effects in normal tissues. In the case of DCA, the main side effect reported was a certain dose‐dependent reversible peripheral neuropathy.^50^ No side effects have been reported when the phenformin is used as antitumour therapy.

Regarding all the results obtained, it is important to note that we demonstrated that BMs decreased cell proliferation and survival, even when used alone, having the potential to be used as an alternative therapy in glioma, as they are able to cross the brain‐blood barrier (BBB).^29^, ^51^ Even with some controversy on this issue regarding the biguanide class, a recent report showed some BBB permeability to these compounds.^52^

This study aimed to demonstrate the existence of new therapeutic approaches, based on cancer altered metabolism, to improve the available therapies. We demonstrated that the different cell lines used in this work presented different responses to TMZ. Transfection with the HOXA9 gene of the human immortalized astrocytes hTERT/E6/E7 cells induced an increase in resistance to TMZ treatment and a more aggressive behaviour.^27^ As we observed in our results, this cell line presents an increased resistance to TMZ comparatively to the glioma cells, what can be due to one of the most common mechanism of resistance in gliomas, the increase in MGMT enzyme activity.^10^ In contrast, the glioma cell lines present a higher sensitivity to treatment, with lower IC_50_ values, probably due do their low/null expression of MGMT.^30^, ^31^ For that reason, we pre‐treated all the cell lines with BMs, in an attempt to modulate metabolism to overcome the resistance demonstrated by these cells. We observed that pre‐treatment with all BMs decrease the initial TMZ IC_50_ values, as well as migration and colony formation ability of cells treated with TMZ, potentiating its effect, particularly in the most resistant cell line expressing MGMT. However, there is no report describing the energy dependence of MGMT enzymatic activity; therefore, other mechanism should be involved. The use of BMs could be a double‐edge sword, as they can induce an alteration of pHe. Some authors showed that the activity of TMZ or the derived metabolites, namely the active metabolite 3‐methyl‐(triazen‐1‐yl) imidazole‐4‐carboxamide (MTIC) seems to be pH dependent (pH > 7). However, there is some controversy on their mechanism of action and additionally, the reports did not specify the place where this activation occurs, inside or outside of tumour cells.^53^, ^54^ Therefore, the activity and subsequent toxicity of MTIC could be altered with the pH gradient, what can explain the chemosensitization of cells when pre‐treated with the BMs. Additionally, pre‐treatment with different BMs was also able to potentiate the TMZ action in glioma cell lines, probably due to other mechanism unrelated to MGMT. Additionally, and supporting our findings, other reports showed the advantage of using these kind of compounds in a combined way with conventional drugs. In fact, Koukourakis et al^55^ sensitized glioblastoma cell lines, namely the most resistant ones, to conventional therapies (radiotherapy and chemotherapy with TMZ), using the glycolytic inhibitor 2‐DG and the LDHA inhibitor oxamate, as well as LDHA gene silencing. Velpula et al^56^ also demonstrated that other mechanisms than MGMT expression can be involved in MDR phenotype, namely in glioblastomas. The authors observed that DCA increases TMZ cytotoxicity, reverting the Warburg effect through tyrosine kinase signalling, namely via EGFRvIII.

Deprivation of tumour energy may predictably potentiate conventional chemotherapeutic treatments, as many of the proteins associated with the MDR phenotype are energy dependent, such as the ATP‐binding cassette (ABC) transporter family responsible for the efflux of different high affinity substrates, namely a wide range of antitumour drugs.^57^ In fact, some reports showed the overexpression of these resistance proteins either in BBB or in glioma cells, preventing TMZ cytotoxicity.^58^, ^59^, ^60^ By Western blot, we demonstrated a strong expression of Pgp in glioma cell lines used in this study (U251 and SW1088) compared to the hTERT/E6/E7 HOXA9 cells (Figure S5). However, this is not the case for U251 cells treated with phenformin, where we observed an increase in ATP intracellular content. Nevertheless, phenformin was also able to alter cancer characteristics and to potentiate the cytotoxicity of TMZ, what can be probably explained by other mechanisms. In fact, the MDR phenotype involves different and complex mechanisms that can be used as target to overcome the low efficacy of employed treatment regimens, and metabolic inhibition could be one of the strategies that can be used for this purpose.

Collectively, our findings provided new insights into cancer cell metabolism as a promising therapeutic strategy for patients with gliomas, increasing therapeutic sensitivity. The use of different metabolic inhibitors combined to standard therapy used in clinical routine, reducing therapeutic doses and consequently decreasing adverse effects in normal brain, could be a new helpful option.

## CONFLICT OF INTEREST

The authors confirm that there are no conflicts of interests.

## AUTHOR CONTRIBUTIONS

DT‐V participated in the conception, design and writing of the manuscript, acquisition, analysis and interpretation of data, as well as development of methodology; SG performed some of the in vivo assays; OQ and FB participated in the conception of the study, data interpretation, and revision of the manuscript and work supervision.
