# Supplementary material for: Bioenergetic modulators hamper cancer cell viability and enhance response to chemotherapy
Source: J Cell Mol Med. 2018 May 29;22(8):3782–94. doi: 10.1111/jcmm.13642 (PMC6050502; doi:10.1111/jcmm.13642)
Supplement: Supplementary file 1 [file JCMM-22-3782-s001.pdf]

Supporting information

Table 1: Measure of extracellular pH in all three cell lines (U251, SW1088 and hTERT/E6/E7 HOXA9) in the presence or absence of the BMs IC_{50} values, after the respective time of incubation. The ΔpH is represented by the difference between the extracellular pH after treatment subtracted the extracellular pH without treatment. Results represent the mean ± SEM of triplicates from at least three independent experiments.

	**Medium w/o BM (48h)**	**Medium w/ DCA (48h)**	**ΔpH**	**Medium w/o BM (72h)**	**Medium w/ 2-DG (72h)**	**ΔpH**	**Medium w/ phenformin (72h)**	**ΔpH**
**U251**	5.7 ± 0.4	6.7 ± 0.4	1.0	5.5 ± 0.1	6.5 ± 0.2	1.0	5.6 ± 0.2	0.1
**SW1088**	6.0 ± 0.4	6.0 ± 0.2	0.0	5.8 ± 0.3	6.3 ± 0.1	0.5	5.7 ± 0.1	-0.1
**hTERT/E6/E7 HOXA9**	5.9 ± 0.3	6.5 ± 0.4	0.6	5.7 ± 0.2	6.6 ± 0.1	0.9	5.8 ± 0.2	0.1

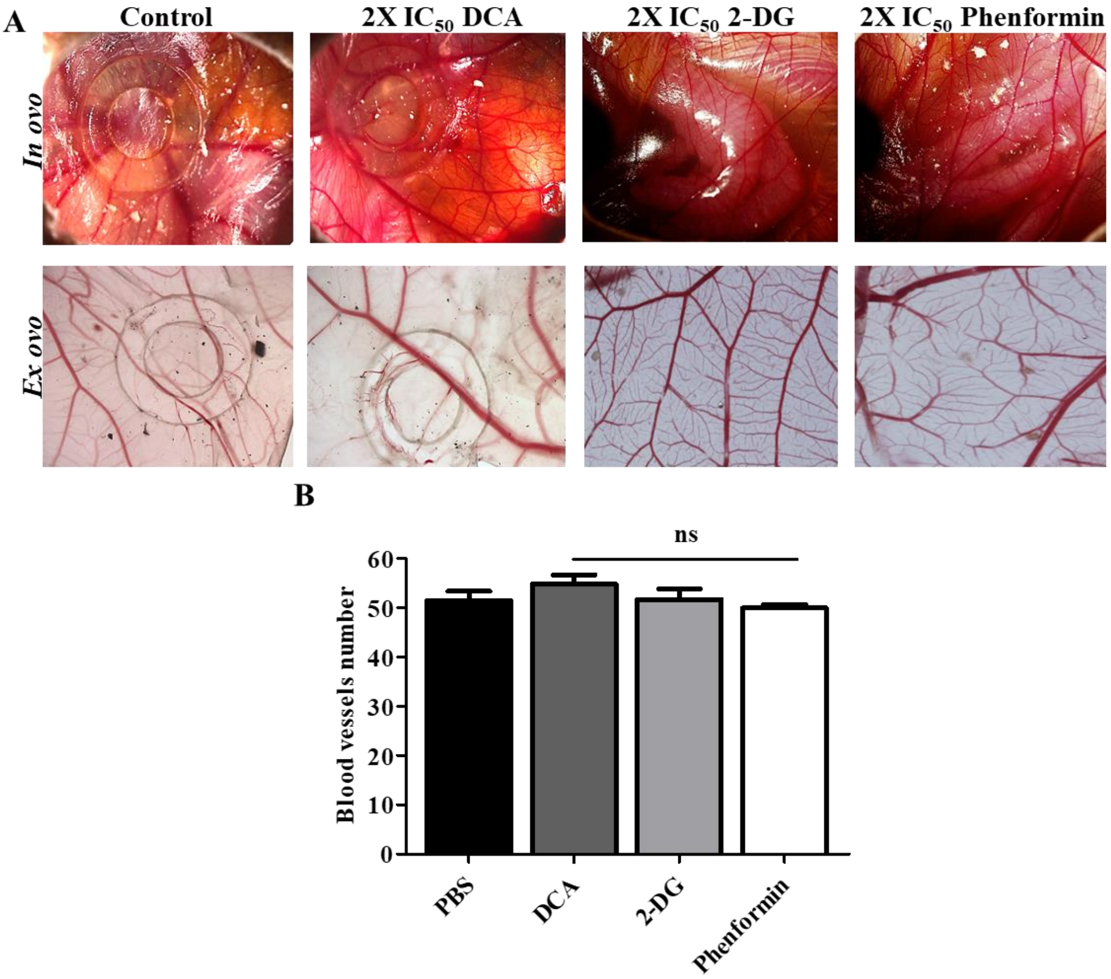

Figure 1: *In vivo* effect of BMs in CAM development. Representative pictures (16× [up] and 12.5× [down] magnifications) of BMs effect on CAM, after 4 days of treatment *in* and *ex ovo*. Representative pictures of BMs effect on the vascularization of CAM (A). Blood vessel quantification in *ex ovo* (B) (control group n=20 eggs; treated group n=20 eggs). Ns: no significant.

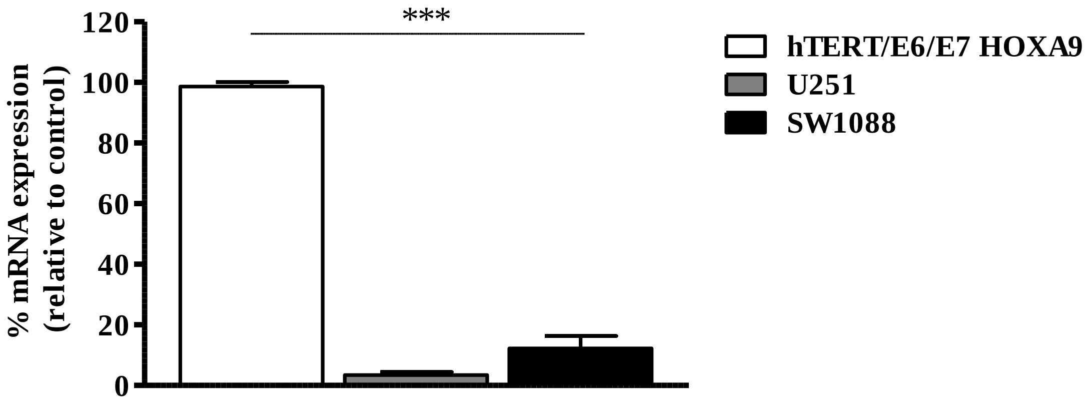

Figure 2: Expression of MGMT gene in hTERT/E6/E7 HOXA9 and glioma cell lines, U251 and SW1088 by quantitative real-time. Results represent the mean ± SD of 3 independent experiments. *** $P< 0.001$, compared to the cells with high gene expression.

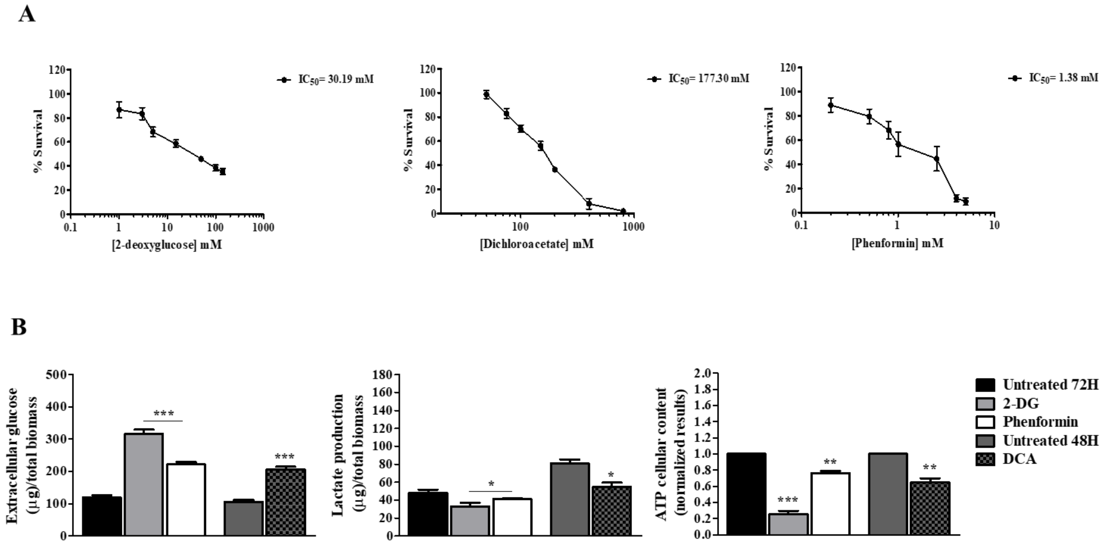

Figure 3: Effect of 2-DG, DCA and phenformin on total biomass (A) and on metabolic profile (B) in hTERT/E6/E7 HOXA9 cells. (A) The cell survival was assessed by sulforhodamine B assay and the IC_{50} was determined. (B) Cells were incubated in the presence of the IC_{50} of the BMs, at the respective time of incubation. After this time, the metabolic parameters were quantified. Glucose and lactate levels were normalized against the biomass content. Untreated cells were used as control. ATP levels were normalized against the protein content of the extract, and against the value obtained

with untreated cells, set as 1. Results are presented as mean ± SD of at least three independent experiments. *$P < 0.05$; **$P < 0.01$; ***$P < 0.001$ compared to untreated cells (control). Ns: no significant.

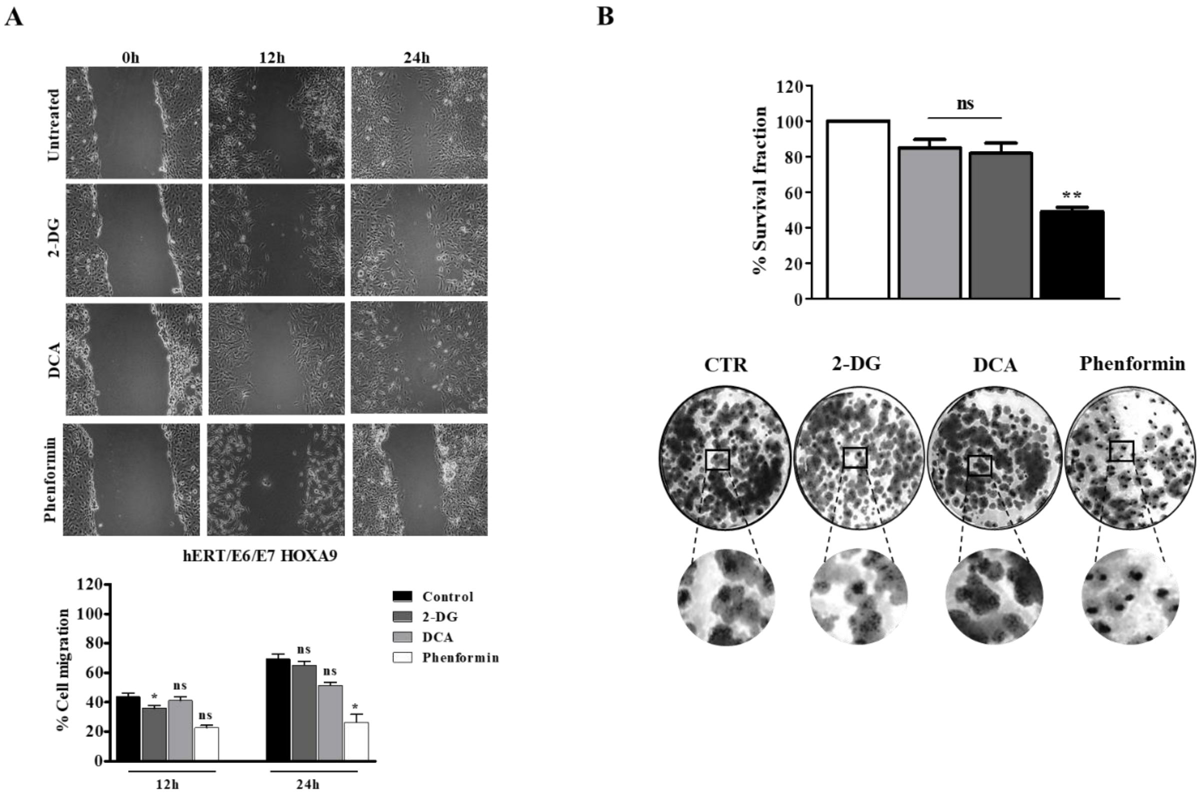

Figure 4: Cell migration (A) and cell colony formation (B) in hTERT/E6/E7 HOXA9 cells, after treatment with BMs 2-DG, DCA and phenformin IC_{50} values. (A) Cell migration was quantified by the wound-healing assay. Pictures were taken at 40x magnification in a Nikon eclipse TE 2000-U microscope. (B) Representative pictures of colony formation. Cells were incubated with different BMs, and after a period of recovery for ten days without compounds, the survival fraction was calculated. Pictures were taken at 200x magnification in a Nikon eclipse TE 2000-U microscope. Results represent the mean + SD of at least 3 independent experiments. *$P < 0.05$; **$P < 0.01$ compared to untreated cells (control). Ns: no significant.

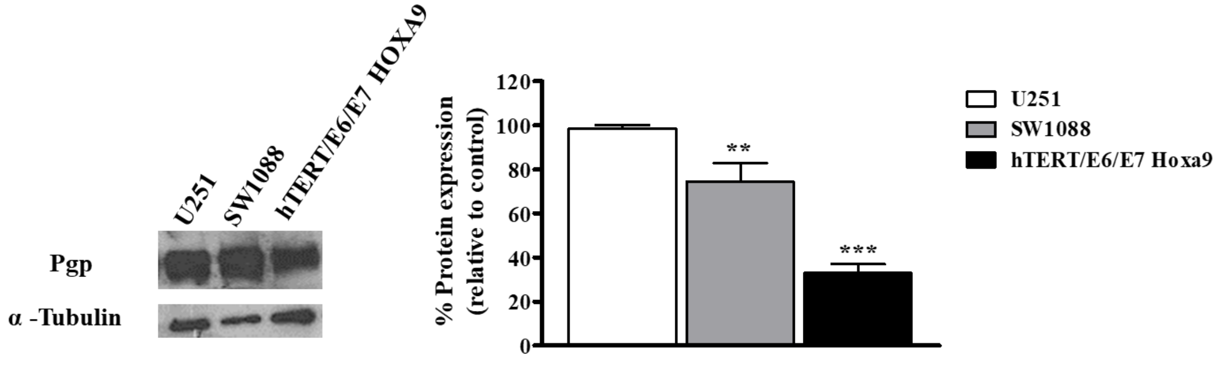

Figure 5: Protein levels of ABC transporter Pgp. Protein levels in breast cancer cells was assessed by Western blot. Results are expressed as mean ± SD of triplicates from three independent experiments. $^{**}P < 0.01$; $^{***}P < 0.001$ compared to the cells with high expression of Pgp.
